# Considerations on the Applicability of Test Methods for Mechanical Characterization of Materials Manufactured by FDM

**DOI:** 10.3390/ma13010028

**Published:** 2019-12-19

**Authors:** Amabel García-Domínguez, Juan Claver, Ana María Camacho, Miguel A. Sebastián

**Affiliations:** Department of Manufacturing Engineering, Universidad Nacional de Educación a Distancia (UNED), 28040 Madrid, Spain; agarcia5250@alumno.uned.es (A.G.-D.); jclaver@ind.uned.es (J.C.); msebastian@ind.uned.es (M.A.S.)

**Keywords:** additive manufacturing, FDM, ABS, anisotropy, infill density, layer orientation, ASTM D638–14:2014, ISO 527–2:2012

## Abstract

The lack of specific standards for characterization of materials manufactured by Fused Deposition Modelling (FDM) makes the assessment of the applicability of the test methods available and the analysis of their limitations necessary; depending on the definition of the most appropriate specimens on the kind of part we want to produce or the purpose of the data we want to obtain from the tests. In this work, the Spanish standard UNE 116005:2012 and international standard ASTM D638–14:2014 have been used to characterize mechanically FDM samples with solid infill considering two build orientations. Tests performed according to the specific standard for additive manufacturing UNE 116005:2012 present a much better repeatability than the ones according to the general test standard ASTM D638–14, which makes the standard UNE more appropriate for comparison of different materials. Orientation on-edge provides higher strength to the parts obtained by FDM, which is coherent with the arrangement of the filaments in each layer for each orientation. Comparison with non-solid specimens shows that the increase of strength due to the infill is not in the same proportion to the percentage of infill. The values of strain to break for the samples with solid infill presents a much higher deformation before fracture.

## 1. Introduction

Currently, the characterization of parts obtained by additive manufacturing (AM) technologies is a very prolific research field. This fact is a consequence, not only of the significant increase of the presence and the importance of additive technologies in a wide range of industries, but it is also due to the lack of specific standardization, as claimed in previous works such as the one by Rodríguez-Panes et al. [1] and in the review made by Popescu et al. [2], who specifically demanded that test standards for Fused Deposition Modelling (FDM) parts should be developed, including the definition of printing parameters such as layer thickness, perimeters, and raster dimensions. Thus, the most commonly applied international standards for tensile tests are ISO 527–2 [3] and ASTM D638–14 [4], but, in both cases, the guidelines provided respond to the characteristics of plastic injection parts, which represents a very different start context. Contrary to the continuity and isotropy of injected plastics, the layer by layer structure of the pieces obtained by additive manufacturing is discontinuous and anisotropic, so the polymeric nature of the material is the only common feature for both technologies. It is also worth mentioning that, while ISO 527–2 and ASTM D638–14 are identified as the consultation documents for tensile tests by the main standards focused on additive manufacturing technologies, as ISO 17296–3:2014 [5] and ISO/ASTM 52921:2013 [6] are, the Spanish standard UNE 116005:2012 [7] has also been considered; this standard is only focused on tensile tests and polymer materials, and it is based on ISO 527–2. Despite both standards being similar, UNE 116005:2012 is developed as a specific standard for additive manufacturing, and it is not a standard recovered from other productive sectors. The national standards must follow the guidelines of the international ones and act as complementary information sources to guide researchers and professionals in specific tasks of applicative character, as in this case, with performing mechanical tests for additive manufacturing purposes.

The experiences developed by researchers when applying these standards in additive contexts have a key role, since the obtained results help to clarify the applicability or not of these standards in different additive scenarios, as well as to provide action guidelines. A significant variety of approaches have been faced in order to determinate the influence of manufacturing parameters on the mechanical response of additive products; for example, ABS specimens for unmanned aerial systems produced by FDM were characterized under tension [8] and under compression [9]; Banjanin et al. [10] evaluated the mechanical response of specimens of polylactide (PLA) and acrylonitrile styrene butadiene (ABS), concluding that the results in ABS showed a higher repeatability under tension than under compression, and vice versa. Chacón et al. [11] assessed the effect of build orientation, layer thickness, and feed rate on the mechanical performance of PLA specimens, stating that, for optimal mechanical performance, low layer thickness and high feed rate values are recommended. In the work of Tanikella et al. [12], a set of seven materials has been tested for determination of tensile strength of FDM specimens fabricated with an open-source 3D printer, concluding that this property highly depends on the mass of the specimen. The influence of the type of infill pattern and density, printing temperature, among other parameters on selected mechanical properties of PLA and ABS samples, were evaluated by Ćwikła et al. [13], showing that, if the maximum strength is the priority, shell thickness should be increased. In the work of Kuznetsov et al. [14], the influence of geometric process parameters on the strength of the sample was also evaluated proposing a new methodology, and concluding that the best combinations of printing parameters allows for obtaining interlayer cohesion close to the one of the feedstock material. Rajpurohit and Dave found a close relationship between the raster angle and failure mode [15]. In addition, Zaldivar et al. [16] investigated the effect of the build orientation on the mechanical properties of a polyetherimide thermoplastic blend as material, concluding that FDM materials behave more like composite structures than isotropic cast resins and that designs should define the build configurations allowable.

**Figure 1:**
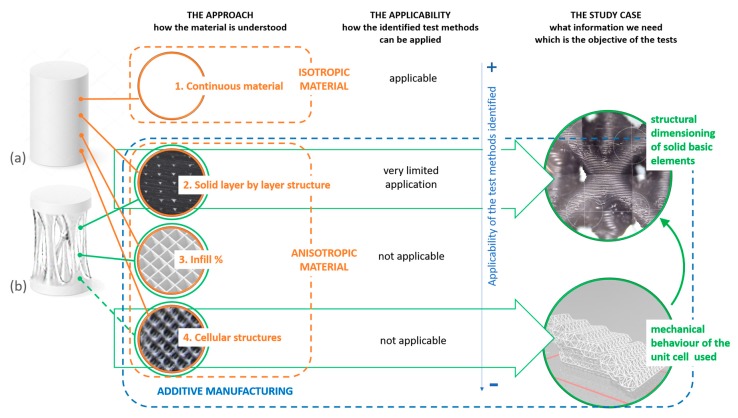
Applicability of conventional test methods for material characterization of parts obtained by additive manufacturing techniques considering their internal morphology: (**a**) basic geometry suitable both for traditional and additive manufacturing technologies. (**b**) complex geometry suitable for additive manufacturing technologies.

However, sometimes, a complementary and previous question is needed because the kind of part we want to produce or the purpose of the data we want to obtain from the tests can guide the definition of the most appropriate specimens. Figure 1 tries to express this idea graphically. A basic geometry usually obtained as a continuous solid by plastic injection (Figure 1a) can be materialized in very different ways when considering additive manufacturing. This includes:

- a layer by layer solid configuration (Figure 1(2)), which means an infill percentage of 100%,
- configurations with different infill percentages (Figure 1(3)),
- and cellular structures (Figure 1(4)).

On the other hand, for more complex geometries that are not possible to manufacture with plastic injection processes but are possible in additive scenarios (Figure 1b), these different approaches could be chosen as alternatives for the materialization of the parts which traditionally would be understood as solid. The differences between the internal material continuity that the selection of one or another of these approaches introduces are significant. Thus, this selection implies different ways of understanding the material since, although in every case mentioned before the polymer used can be the same, the behavior of the piece is highly conditioned by internal morphology of those parts traditionally understood as solid.

As exposed before, from the point of view of the applicability of the mentioned standards, only a continuous material could be characterized. However, it is interesting to reflect the different levels of applicability that the selection of each approach introduces in the context of additive processes. In that sense, the applicable criterion is the continuity or not of the internal morphology of the piece. Thus, a solid layer by layer structure would represent the closest conditions to the ones considered in these standards. In the case of predefined infill patterns, these standards are not really applicable, but, in practice, many studies use this kind of specimens and analyze their behavior by applying them. Finally, cellular structures are similar to these infill patterns in terms of discontinuity, but this group includes lattice structures and approaches in which the sizing of some parts or elements can change along the piece and those fluctuations provide graded densities.

This way, the design of each case study makes each approach more or less appropriate, and it is essential to take it into account. In that sense, the methodology optimization developed by the authors in a previous work [17] and its data needs can be a good example. The mentioned methodology uses optimized lattice structures for the infill areas where each bar is sized according to the stress it can withstand [18]. In order to calculate the section needed in each case, values of the material strength are requested by the structure of the methodology. Since these bars have diameters between 1 and 2.5 mm, infill patterns or cellular structures cannot be implemented into them, so the layer by layer solid configuration is the only option. In addition, as each bar has a different section as a consequence of the different stress values calculated, the lattice structure cannot be considered as uniform along the piece. Thus, those approaches that estimate the mechanical behavior of cellular structures through the testing of specimens out of the standards and that contain a certain number of unit cells would not be of application in this particular case, as in the works of Chen et al. [19], who developed a finite element mesh based method for optimization of lattice structures for AM; Hussein [20], who studied the development of lightweight cellular structures for additive manufacturing with metal; Mahmoud and Elbestawi [21], where lattice structures were fabricated by additive manufacturing in orthopedic implants; Maliaris and Sarafis [22], where lattice structures were modelled using a generative algorithm; Panda [23] and Weeger et al [24], who worked in the design and development of cellular structures for AM; or Vannutelli [25], where the mechanical behavior of lattice structures was analyzed. As Figure 1 exposes, a solid layer by layer configuration represents the most consistent alternative with the nature of this particular case study and the data that are needed. This way, the identified standards could be applied, always considering their limitations in additive contexts.

Accordingly, the aim of this work is to analyze the mechanical behavior of solid structures manufactured by FDM (as a typology of interest in different design contexts supported by additive technologies, as the ones described above) compared to non-solid ones, and to determine which standard provides better results for the mechanical characterization of materials manufactured by FDM, giving some guidelines in the application of them due to the lack of specific standards for characterization of materials manufactured by FDM. To achieve this goal, a series of tensile tests are carried out using solid specimens obtained by FDM, and according to the specifications of UNE 116005:2012 (based in ISO 527–2) and ASTM D638–14. The obtained results are interpreted together with the layer structures observed in the specimens after fracture.

## 2. Materials and Methods

### 2.1. Work Methodology

**Figure 2:**
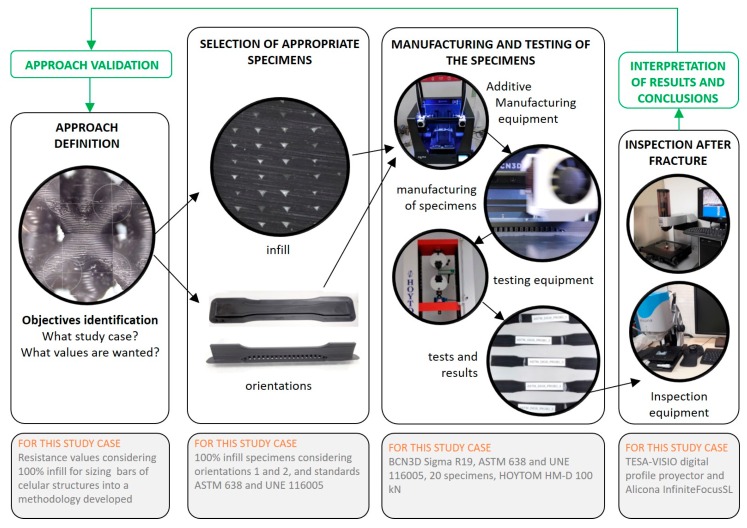
Approach definition based in Figure 1 and sketch of the work methodology.

According to the aim of this work, and in order to have a clear view of the research methodology followed in this work, Figure 2 shows the approach definition based in the different situations presented in Figure 1, and a summary of the main steps of the methodology. Four main steps are established, and then basic information related to the particular context of this work is indicated for each one of these stages. As shown in the work diagram, at the end of the process, the obtained results and the conclusions derived from them must be analyzed in order to assess whether the results fit the objectives of the study. The main aspects of the work developed in these stages are exposed in the following sections. Afterwards, the results are compared with the ones obtained by the authors in a previous work in which the specimens used were fabricated using infill patterns [26].

### 2.2. Materials and Equipment

**Figure 3:**
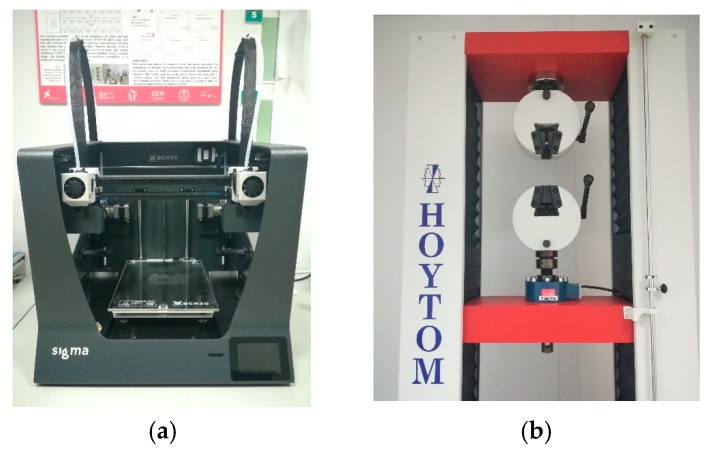
Equipment for additive manufacturing and testing of specimens: (**a**) a dual extruder BCN3D R19 printer; (**b**) detail of the working area in Hoytom HM-D 100kN Universal testing machine.

The material used in this work is ABS Pro of the commercial house BCN3D (Castelldefels, Spain), whose main physical and mechanical properties are presented in Table 1. The FDM printer to be used is a dual extruder BCN3D R19 printer (BCN3D, Castelldefels, Spain), presented in Figure 3a, whose main technical characteristics are shown in Table 2. The software Cura (Ultimaker, Geldermalsen, The Netherlands) is typically used to export the three-dimensional models of the specimens to G-code. In this paper, the version BCN3D Cura 2.1.4 adapted by BCN3D (Castelldefels, Spain) has been used. Figure 3b shows the equipment for the mechanical testing, a universal testing machine (model Hoytom HM–D 100 kN, Hoytom, S.L., Leioa, Spain).

**Figure 4:**
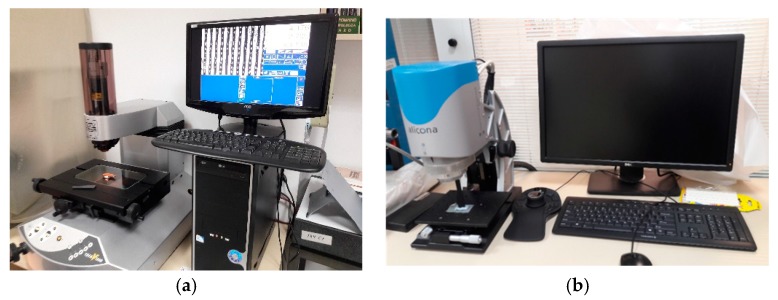
Equipment for surface analysis: (**a**); digital profile projector TESA-VISIO; (**b**) optical micro-coordinate measurement machine IF-SL ALICONA.

For analysis of the surface, a digital profile projector TESA-VISIO (TESA SA, Renens, Switzerland) and an optical micro-coordinate measurement machine IF-SL ALICONA (Bruker Alicona, Graz, Austria) are used (see Figure 4).

### 2.3. Printing Parameters and Definition of a Work Plan

The general manufacturing parameters for the FDM printing of the specimens are presented in Table 3.

**Figure 5:**
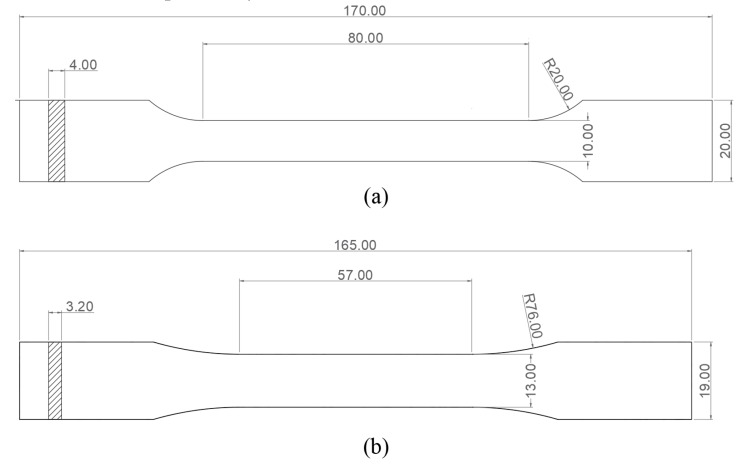
Geometry of the specimens according to different international standards: (**a**) Type 1A: UNE 116005 [7]; (**b**) Type I: ASTM D638–14 [4].

Due to the lack of specific standards for material characterization of FDM parts, as explained before, two different standards are going to be used for mechanical behavior testing: UNE 116005:2012 (based in ISO 527–2) [7] and ASTM D638-14:2014 [4]; the geometry of the specimens according to both standards is shown in Figure 5, where Type 1A and Type 1 are the geometries chosen for both standards, respectively.

**Figure 6:**
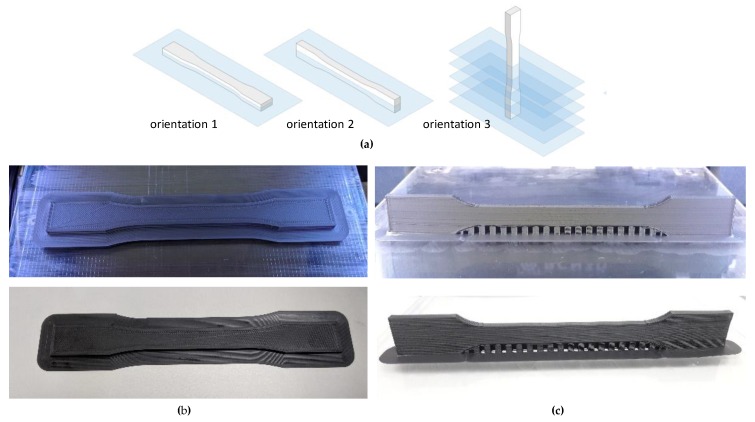
(**a**) typical build orientation of the samples; (**b**) specimen printed with orientation 1; (**c**) specimen printed with orientation 2.

As explained by Rodríguez-Panes et al. [26], the build orientation of the test specimen is one of the most influencing parameters on the mechanical properties of FDM parts. Figure 6a presents the three possible orientations, orientation 1 (flat) and 2 (on-edge) being the ones chosen in this work since orientation 3 is not appropriate for tensile testing due to the arrangement of the filaments perpendicular to the direction of the load. Figure 6b and c shows the two orientation of the samples used in this work.

Five specimens for each orientation (1 and 2) are going to be tested according to either standard UNE 116005:2012 and ASTM D638–14:2014. A summary of the experimental work plan and nomenclature used in experiments is presented in Table 4.

Results from the experimental testing of specimens in Table 4 are going to be compared to those ones obtained ii [26] in specimens with non-solid infill; the material of the experiments was ABS (PrintedDreams) in blue and the test procedure according to standard ASTM D638–14:2014 with type I general usage test specimen. The aim is to observe the influence of the infill in the characterization of materials obtained by FDM and for validation purposes. A summary of the cases for comparison is gathered in Table 5.

### 2.4. Experimental Procedure for Tensile Testing

**Figure 7:**
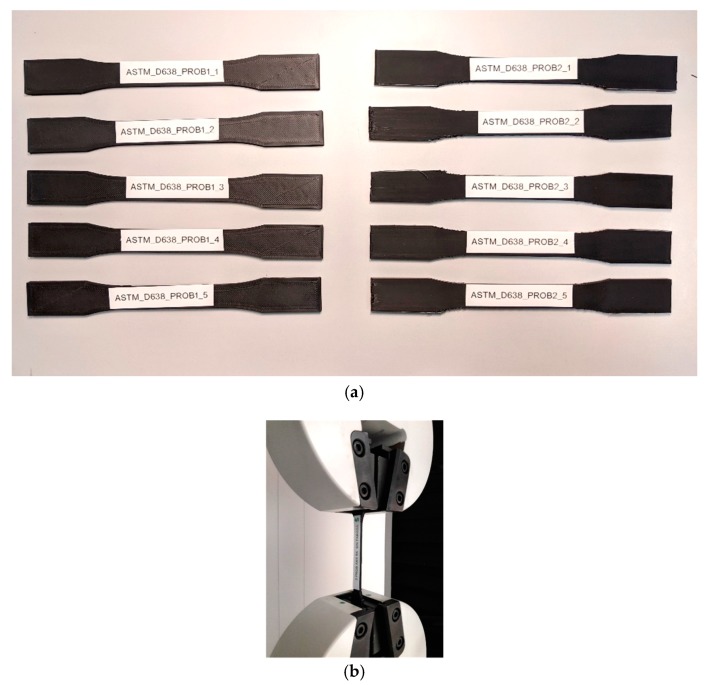
(**a**) the test specimens manufactured according to ASTM D638–14; (**b**) specimen placement before tensile testing.

The specimens in Table 4 are printed according to printing parameters in Table 3. Figure 7a shows the group of samples according to ASTM D638–14:2014. Each specimen is fixed by the grips as it is seen in Figure 7b, showing a specimen just before tensile testing.

The tests are performed at a velocity of 5 mm/min until breakage. The mechanical properties to be obtained are: tensile strength at yield (*σ*_y_), tensile strength at break (*σ*_U_) and nominal strain at break (ε_t_). Equations (1) to (3) show how these parameters can be obtained, as defined in the standard ASTM D638-14 [4]:

(1) $$\sigma_{y}=\frac{F_{max}}{A_{0}}$$

(2) $$\sigma_{U}=\frac{F_{break}}{A_{0}}$$

(3) $$\varepsilon_{t}\left(\%\right)=\frac{\Delta L}{L_{0}}\times 100$$

being:

*F_max_*: the maximum force sustained by the specimen,
*F_break_*: the force sustained by the specimen at breakage,
*L_0_*: original grip separation,
Δ*L*: extension (change in grip separation).

## 3. Results and Discussion

### 3.1. Mechanical Behavior of Solid Specimens

**Figure 8:**
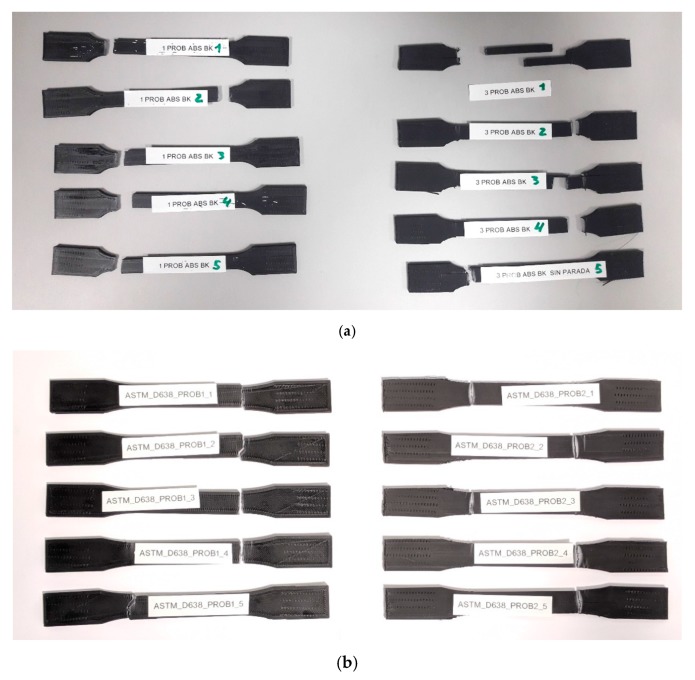
Specimens after tensile testing: (**a**) according to UNE 116005:2012; (**b**) according to ASTM D638–14:2014.

The specimens after tensile testing are presented in Figure 8. Most of them break in close to the radius of fillet close to the grips, which is common for FDM specimens [27], and it is due to stress concentrations at fillet areas [10], being the kind of fracture brittle, with no plastic deformation observed. As Wu et al. reported [28], craze is the main plastic deformation mechanism of ABS, and a great number of crazes are generated perpendicular to the load direction. This brittle behavior is typical in FDM parts, and it has been explained by other authors due to the presence of voids that help the crack initiation and propagation resulting in abrupt rupture [15]; the presence of voids will be analyzed further in Section 3.2.

**Figure 9:**
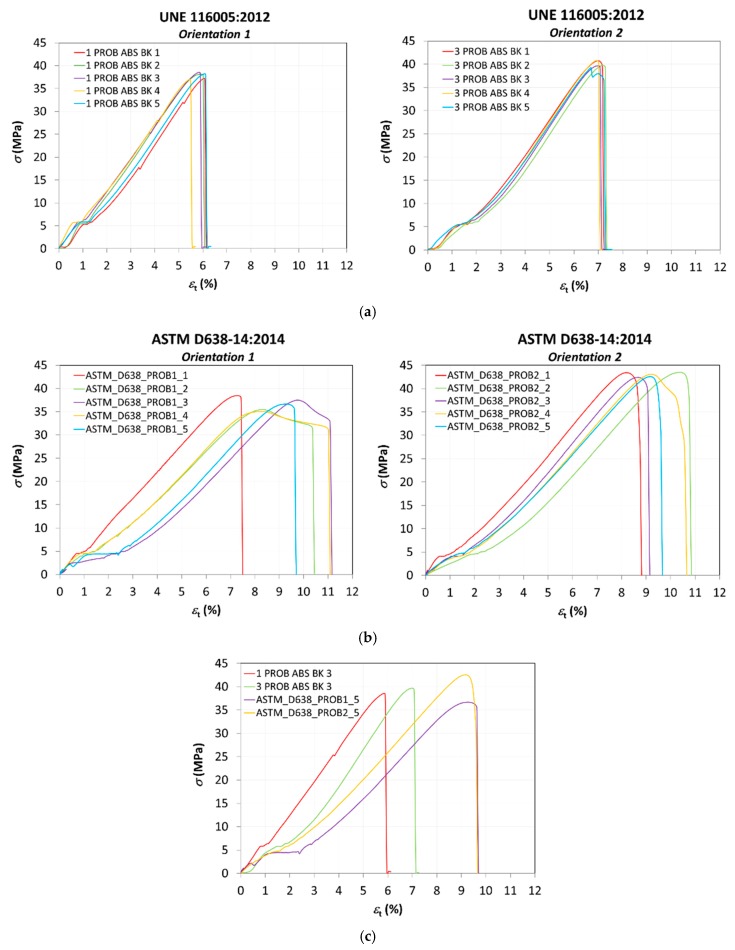
Stress–strain curves. (**a**) according to UNE 116005:2012; (**b**) according to ASTM D638-14; (**c**) comparison of results with the intermediate values of each series of tests.

Nominal stresses and strains are obtained for all the tests performed and presented in Figure 9 as stress–strain curves. Figure 9a presents the results according to the standard UNE 116005:2012 for printing orientations 1 and 2; and Figure 9b according to the standard ASTM D638–14, also for both orientations. A comparison of results with the intermediate values of each series of tests is also shown in Figure 9c.

The mechanical properties (nominal strain at break, tensile strength at yield, tensile strength at break,) obtained from the tensile tests are presented in Table 6.

Tests performed according to standard UNE 116005:2012 present a much better repeatability than the ones according to ASTM D638–14. This is a very important finding since the UNE standard is specifically designed for specimens fabricated by additive manufacturing and proves that the geometry used is more appropriate for characterizing materials obtained by FDM than the one used from international standards such as ASTM D638–14, very often used in the scientific literature for these purposes. Except specimen 3 PROB ABS BK 5, all the specimens experience a brittle breakage without plastic deformation. On the contrary, results for ASTM D638–14 show a higher variability, especially for orientation 1, where some specimens exhibit some plastic deformation before fracture (ASTM_D638_PROB1_2, ASTM_D638_PROB1_3 y ASTM_D638_PROB1_4); elongations are particularly higher than the ones obtained by the standard UNE 116005:2012.

**Figure 10:**
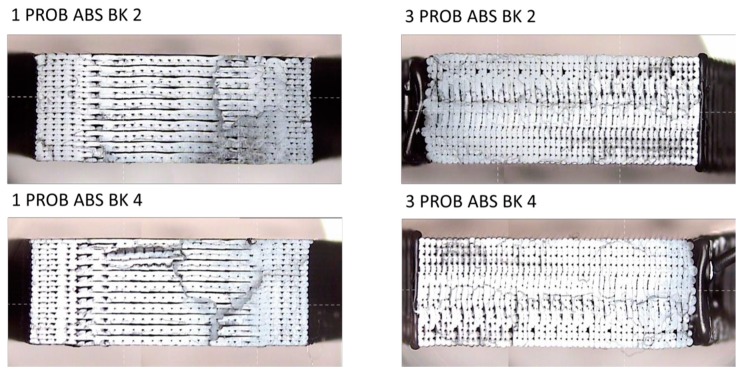
View of the fracture surface of two specimens tested with orientation 1 (**left**) and orientation 2 (**right**) with digital profile projector TESA-VISIO.

As a general trend, orientation 2 provides higher strength to the parts obtained by FDM according to results for both standards, but the influence of the orientation is more important in the case of the standard ASTM. This behavior is coherent with the orientation of the filaments in each layer for orientations 1 and 2, as shown in Figure 10. In this figure, the fracture surface of two of the specimens tested for each orientation (see designation in Figure 8a) is observed, showing the differences in the inner distribution of the filaments as well. These observations are in consonance with the work of Aliheidari et al. [29], who claimed that the nature of the layered structure, and, particularly, the adhesion between the layers, have a direct impact on the mechanical properties.

**Figure 11:**
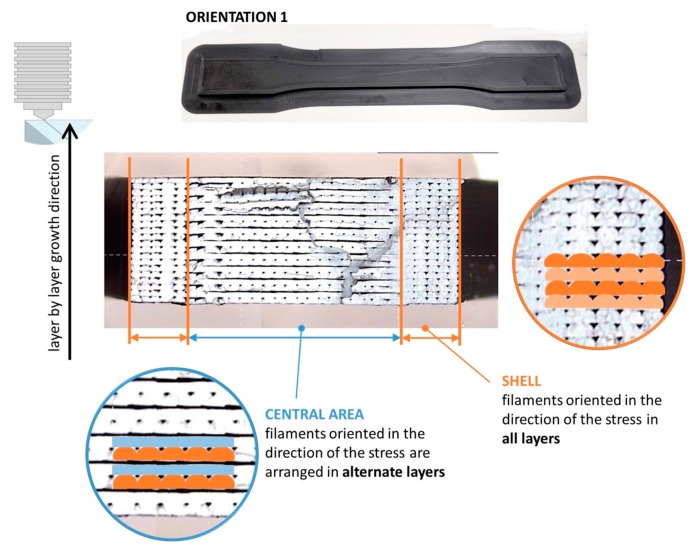
Layout of the filaments related to the longitudinal stress direction for specimens with orientation 1. Identification of central area and shell.

**Figure 12:**
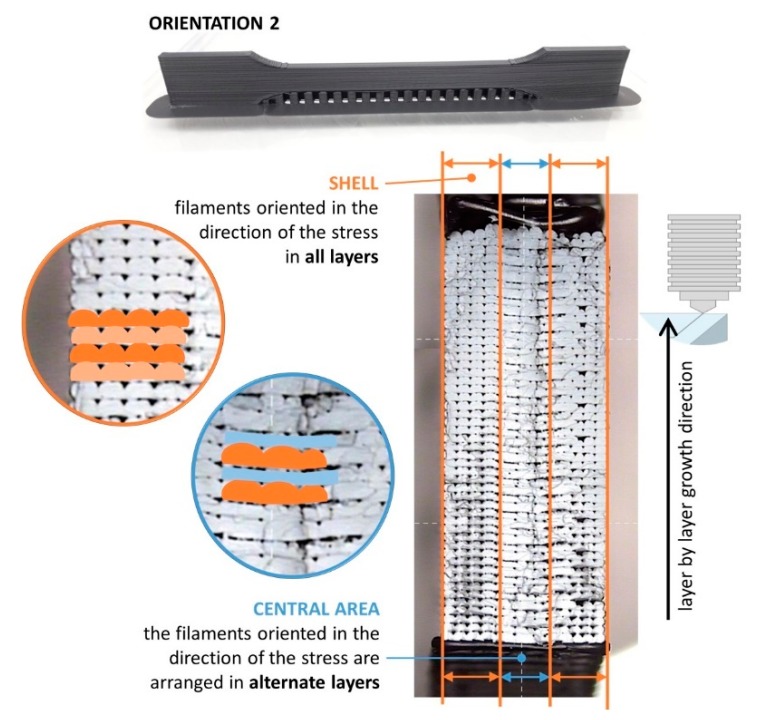
Layout of the filaments related to the longitudinal stress direction for specimens with orientation 2. Identification of central area and shell.

Observing the images in detail and placing the specimens in the direction of vertical growth of layer (Figure 11 and Figure 12), it is possible to distinguish in both cases two lateral areas (shell) where the beads deposited during the printing have the same arrangement in all the layers, being parallel to the longitudinal axis of the specimen and a central area, in the middle of them, where the arrangement of the beads is different in alternate layers, being in one layer the longitudinal direction of the specimen, and in the above and below layers, the transverse direction. The layers in the longitudinal direction benefit from the alignment of polymer molecules along the stress axis [30].

The areas where the filaments are oriented in the direction of the load (shell) are expected to have a higher strength because the filaments contribute together to the strength of the specimen. On the contrary, the central areas present a different behavior: on one hand, there are layers where the filaments are in the same direction than the load, but there are also layers where the filaments are arranged perpendicular to the stress direction, so the cohesion between filaments is crucial to keeping the integrity of the specimen. This situation is analogous to a composite reinforced by continuous or discontinuous fibres, respectively [31]. In this sense, the percentage of section with higher strength provided by the lateral areas (shell), where the filaments are arranged in the same direction than the load, is clearly higher in specimens with orientation 2; this fact justifies their better performance under tension as presented in Figure 9 and Table 6.

**Figure 13:**
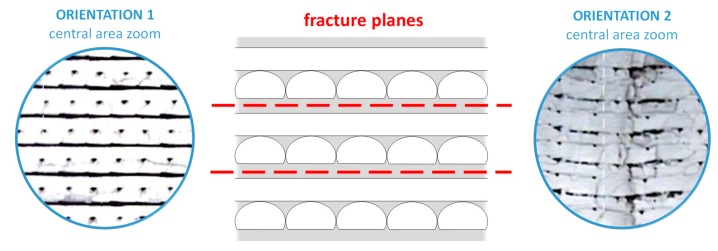
Fracture planes in central areas in specimens with both orientations.

In addition, Figure 11 and Figure 12 allow for appreciating a pattern in the fracture of the specimens with both orientations. Figure 13 shows this situation with more detail. This fibre discontinuity has been reported by other authors [15] as the reason for premature failure of the part, resulting in brittle fracture.

**Figure 14:**
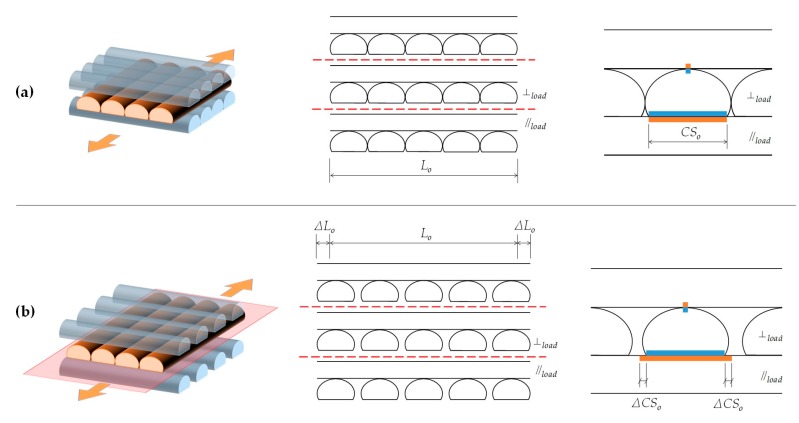
Elongations and displacements between layers. (**a**) state before deformation; (**b**) state after deformation.

Throughout the entire central area of both types of specimens, the loss of cohesion between the layers occurs in the same planes. Thus, as shown in Figure 13, the plane between the bottom of a layer (in which the filaments are in the same direction of the load) and the top of the layer below it (in which the filaments are arranged perpendicular to the load) results in being a weak point of these structures. As it is possible to appreciate in Figure 13, and also in Figure 11 and Figure 12, the cohesion between layers along these planes fails in all cases. In non-solid specimens, the brittle interface fracture leading to delamination between layers in FDM specimens is explained by the weak interlayer bonding or to interlayer porosity, as explained in the work by Ziemian et al. [30], who emphasized that the tensile strength is greatly affected by the fiber to fiber fusion and any air gap between the fibers. Figure 14 tries to illustrate the reason of this recurring behavior in these planes. As shown in Figure 14b, when the deformation of the specimen starts the filaments parallel to the load are the ones that are really affected by the load and so they are the first to be deformed. On the other hand, the bonding between the filaments arranged perpendicular to the load is not strong enough, and it fails easily. 

If the elongation of the filaments oriented in the direction of the load is related to the contact surface between the filaments of adjacent layers, very different situations can be observed. As shown in Figure 14a, before the deformation of the specimen, the contact surface (CS) between two filaments located on adjacent layers is clearly defined. However, when deformation starts (Figure 14b), the filaments in the same direction of the load will suffer certain elongation while the ones arranged perpendicular to the load not. Thus, the dimensions of that initial contact surface become different because the load supported by the filaments of each layer depends on their orientation and the cohesion between filaments of different layers is not strong enough to cause equivalent deformation in alternant layers. 

Considering as the initial reference a layer with the filaments oriented in the same direction of the load and given that they are the ones which mainly support the load applied and which are deformed, Figure 14b also shows as this phenomenon is very different if the upper or lower layer is considered. In the first case, the contact surface is significantly smaller, and once the union between filaments of layers oriented perpendicular to the load has failed, these filaments can move away maintaining their punctual unions with the filaments parallel to the load of the adjacent layers, which are being deformed. Thereby, as shown in Figure 14b, these relative displacements between the contact surfaces of filaments located on adjacent layers become relevant when the lower layer is considered, due to the larger initial contact surface and its different evolution in each layer. For this reason, the fracture planes shown in Figure 13 appear. Recent studies combined computational and experimental techniques [32] to evaluate the cohesive strengths between filaments and seems to be a promising field of research in fracture mechanisms of FDM parts.

### 3.2. Comparative Analysis with Conventional Samples with Pattern Infill

**Figure 15:**
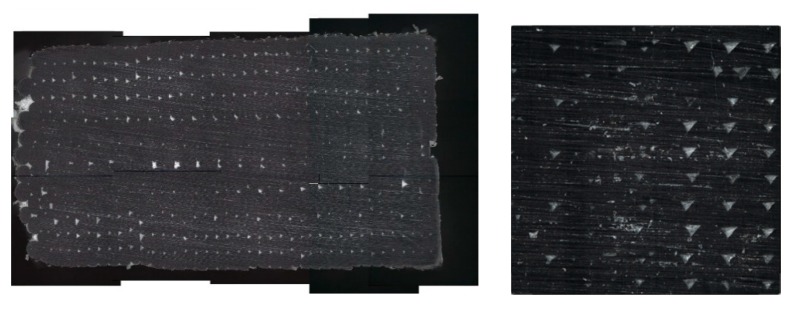
Visualization of the gaps between layers in a specimen performed by an optical micro-coordinate measurement machine IF-SL ALICONA.

Before the comparison is done, it is important to clarify that, although the specimens in this work have been printed as solid parts, the additive manufacturing technique leaves some gaps between layers as presented in Figure 15, so the infill is supposed to be close to 100% infill, but not completely. These triangular air voids are responsible for the decrease of the tensile strength because of a decrease in the cross-sectional area of the specimen [30] compared to bulk materials. Moreover, the lower strength of FDM samples compared with injection-molded samples have been attributed to the gaps between filaments and inner pores within them [28]. 

**Figure 16:**
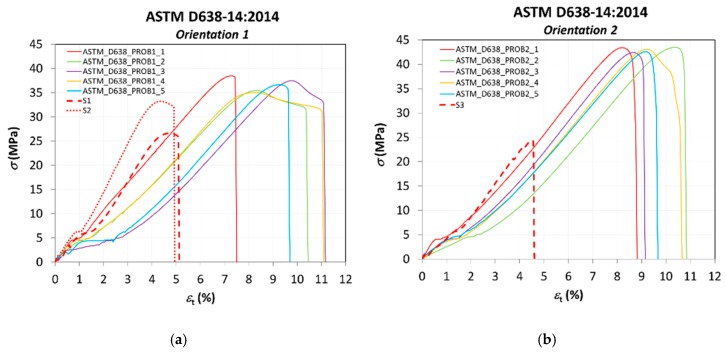
Comparison of results with non-solid specimens (Table 5) tested by Rodríguez-Panes et al. [26]: (**a**) specimens with orientation 1 and 20 and 50% infill; (**b**) specimen with orientation 2 and 20% infill.

A comparison of the results to those ones obtained by Rodríguez-Panes et al. [26], where a pattern infill (non-solid) was used in the FDM specimens, is presented in this section according to Table 5. Comparison is realized with results from standard ASTM D638–14:2014 (Figure 16), as it was the same standard used in the previous work. 

Table 7 presents the data of mechanical properties obtained from graphs of Figure 16.

Tensile strengths of ABS parts obtained by FDM have been reported to be in the range of 11–40 MPa [10]; the explanation to this wide range is associated to their anisotropic behavior. Results are in good agreement with previous works such as the one by Tymrak et al. [33], with tensile strengths around 30 MPa, or the work by Banjanin et al. [10], with an average value of tensile stresses for ABS samples of 31 MPa, considering that they used non-solid specimens, so they are expected to have lower values than the ones obtained in our work with solid ones; in fact, these reference values are in very good agreement with the ones obtained in our previous work with non-solid samples [26], used for comparison in this section.

As expected, for the specimens with an infill percentage of 20% (S1 and S3), the mechanical properties are the poorest ones, the effect of the infill being more significant in the case of orientation 2, where the differences in tensile strength at yield and break are particularly high (almost double). On the other hand, the sample with an infill percentage of 50% (S2) shows a higher strength than samples S1 and S3, but lower than the strength provided by the solid infill of samples ASTM-O1. Nevertheless, the increase of strength due to the infill does not seem to be in the same proportion to the percentage of infill; that is, for an increase of the infill percentage of almost 400% (from 20% to almost 100% infill for solid specimens), the increase of tensile strength is only 37.83%, and, for an increase of infill percentage of 150%, the increase of tensile strength is 25.04%. In the case of the strain to break, the values for the samples with solid infill presents a much higher deformation before fracture.

## 4. Conclusions

In general, tests performed according to the specific Spanish standard for additive manufacturing UNE 116005:2012 present a much better repeatability than the ones according to the general test standard ASTM D638–14, which proves that the geometry of sample and procedure used in the standard UNE is more appropriate for characterizing materials obtained by FDM and for comparison between different materials. All the specimens according to the standard UNE experience a brittle breakage without plastic deformation. Results for ASTM D638–14 show a higher variability, especially for orientation 1, where some specimens exhibit some plastic deformation before fracture and elongations are particularly higher than the ones obtained by the standard UNE.

As a general trend, orientation 2 provides higher strength to the parts obtained by FDM, but the influence of the orientation is more significant in the case of the standard ASTM. This behavior is coherent with the arrangement of the filaments in each layer for each orientation, the percentage of the section with higher strength provided by the lateral areas (shell) being clearly higher in specimens with orientation 2, which justifies their better performance under tension.

Comparison with non-solid specimens (and different percentage of infill) show that, for samples with an infill percentage of 20% (S1 and S3), the mechanical properties are the poorest ones, the effect of the infill being more significant in the case of orientation 2. The sample with an infill percentage of 50% (S2) shows a higher strength than samples S1 and S3, but it is lower than the strength provided by the solid infill of samples, although the increase of strength due to the infill does not seem to be in the same proportion to the percentage of infill. In the case of the strain to break, the values for the samples with solid infill presents a much higher deformation before fracture.

As a general remark and given that national standards follow the guidelines of the international ones (acting as complementary information sources), positive experiences with national standards as the one presented in this work could be considered in future updates of international standards.

## Figures and Tables

**Table 1:** Main mechanical and physical properties of the filaments according to the manufacturer.

Material (Manufacturer)	Color	Tensile Strength (MPa)	Hardness, *HB* (MPa)	Modulus of Elasticity (GPa)	Density (kg/m^3^)
ABS (BCN3D)	Black	45	97	2.3	1040

**Table 2:** Main technical characteristics of the Fused Deposition Modelling (FDM) printer BCN3D R19.

Characteristic	Value
Maximum printing volume (mm^3^)	210(x) × 297(y) × 210(z)
Firmware	BCN3D Sigma – Marlin
Nozzle diameter (mm)	0.3 mm/0.4 mm (Standard)/0.5 mm (Special)/0.6 mm/0.8 mm/1.0 mm
Resolution (mm)	0.05–0.5
Maximum printing velocity (mm/s)	50
Maximum displacement velocity (mm/s)	200

**Table 3:** Manufacturing parameters with FDM printer BCN3D R19.

Material	Extruder Temp. (°C)	Bed Temp. (°C)	Wall/Infill Speed (mm/s)	Layer Height (mm)	Adhesion Platform	Retraction Dist. (mm)/Speed (mm/s)	Wall/Infill Pattern
ABS (BCN3D)	260	90	50	0.1	YES (8 mm)	4/40	Solid

**Table 4:** The experimental work plan and nomenclature of the specimens.

Standard	Orientation 1	Orientation 2
UNE 116005:2012	1 PROB ABS BK 1	3 PROB ABS BK 1
	1 PROB ABS BK 2	3 PROB ABS BK 2
	1 PROB ABS BK 3	3 PROB ABS BK 3
	1 PROB ABS BK 4	3 PROB ABS BK 4
	1 PROB ABS BK 5	3 PROB ABS BK 5
ASTM D638–14:2014	ASTM_D638_PROB1_1	ASTM_D638_PROB2_1
	ASTM_D638_PROB1_2	ASTM_D638_PROB2_2
	ASTM_D638_PROB1_3	ASTM_D638_PROB2_3
	ASTM_D638_PROB1_4	ASTM_D638_PROB2_4
	ASTM_D638_PROB1_5	ASTM_D638_PROB2_5

**Table 5:** Nomenclature of the specimens and printing parameters by Rodriguez-Panes et al. [26].

Nomenclature	Layer Height (mm)	Orientation	Infill (%)
S1	0.1	1	20
S2	0.1	1	50
S3	0.1	2	20

**Table 6:** Mechanical properties and nomenclature of the group of tests.

Group	Nomenclature	ε_t_ (%) Mean (Standard Deviation)	σ_y_ (MPa) Mean (Standard Deviation)	σ_U_ (MPa) Mean (Standard Deviation)
UNE 116005:2012 (Orientation 1)	UNE-O1	5.89 (0.26)	37.82 (0.66)	37.82 (0.66)
UNE 116005:2012 (Orientation 2)	UNE–O2	7.05 (0.11)	39.65 (1.42)	39.64 (1.42)
ASTM D638–14:2014 (Orientation 1)	ASTM–O1	9.87 (1.57)	36.61 (1.42)	33.38 (3.03)
ASTM D638–14:2014 (Orientation 2)	ASTM–O2	9.61 (0.86)	43.00 (0.49)	40.09 (2.38)

**Table 7:** Comparison of mechanical properties in samples with solid (ASTM–O1, ASTM–O2) and non-solid (S1, S2, S3) infill.

Group	Nomenclature	ε_t_ (%)	σ_y_ (MPa)	σ_U_ (MPa)
ASTM D638–14:2014 (Orientation 1)	ASTM–O1	9.87	36.61	33.38
	S1	5.09	26.56	25.83
	S2	4.90	33.21	32.06
ASTM D638–14:2014 (Orientation 2)	ASTM–O2	9.61	43.00	40.09
	S3	4.54	24.38	24.38
